# Frequency-dependent changes in the amplitude of low-frequency fluctuations in patients with Wilson’s disease: a resting-state fMRI study

**DOI:** 10.1007/s11011-016-9946-3

**Published:** 2017-01-24

**Authors:** Xiaopeng Hu, Siyi Chen, Chang-Bing Huang, Yinfeng Qian, Yongqiang Yu

**Affiliations:** 1 Department of Radiology, First Affiliated Hospital of Anhui Medical University, Hefei, 230022 China; 2 Key Laboratory of Behavioral Science, Institute of Psychology, Chinese Academy of Sciences, Beijing, 100101 China; 3 Department Psychologie, Ludwig-Maximilians-Universität, 80802 Munich, Germany

**Keywords:** Wilson’s disease, Resting-state functional magnetic resonance imaging, Amplitude of low-frequency fluctuations

## Abstract

To investigate the frequency-dependent changes in the amplitude of low-frequency fluctuations (ALFF) in patients with Wilson’s disease (WD). Resting-state function magnetic resonance imaging (R-fMRI) were employed to measure the amplitude of ALFF in 28 patients with WD and 27 matched normal controls. Slow-5 (0.01–0.027 Hz) and slow-4 (0.027–0.073 Hz) frequency bands were analyzed. Apart from the observation of atrophy in the cerebellum, basal ganglia, occipital gyrus, frontal gyrus, precentral gyrus, and paracentral lobule, we also found widespread differences in ALFF of the two bands in the medial frontal gyrus, inferior temporal gyrus, insula, basal ganglia, hippocampus/parahippocampal gyrus, and thalamus bilaterally. Compared to normal controls, WD patients had increased ALFF in the posterior lobe of the cerebellum, inferior temporal gyrus, brain stem, basal ganglia, and decreased ALFF in the anterior lobe of the cerebellum and medial frontal gyrus. Specifically, we observed that the ALFF abnormalities in the cerebellum and middle frontal gyrus were greater in the slow-5 than in the slow-4 band. Correlation analysis showed consistently positive correlations between urinary copper excretion (Cu), serum ceruloplasmin (CP) and ALFFs in the cerebellum. Our study suggests the accumulation of copper profoundly impaired intrinsic brain activity and the impairments seem to be frequency-dependent. These results provide further insights into the understanding of the pathophysiology of WD.

## Background

Wilson’s disease (WD) or hepatolenticular degeneration, is first defined by Alexander Kinnier Wilson in 1912 (Wilson 1912). The physical burden of the disease falls largely in the liver and brain (Reilly et al. 1993). Accumulation of cooper in the liver can lead to elevated serum aminotransferases, chronic hepatitis, and cirrhosis (Ala et al. 2007). In the brain, most copper is deposited in the basal ganglia, particularly in the lenticular nucleus, which areas normally participate in action selection, movement control, procedural and reward learning, and emotion regulation (Yamada et al. 2004; Turner et al. 2003; Packard and Knowlton 2002; Radua et al. 2010). About half the people with Wilson’s disease will develop neurological or psychiatric symptoms, such as mild cognitive deterioration in the early stage (de Bie et al. 2007), and dysarthria, abnormal gait, risus sardonicus, dystonia, rigidity, bradykinesia in the late stages (Machado et al. 2006), indicating potential brain changes in WD patients. Previous studies have revealed generalized brain atrophy, lesions in the brainstem, cerebellum, basal ganglia, subcortical white matter, midbrain, and pons of WD patients with conventional magnetic resonance imaging (MRI) (Page et al. 2004; Sinha et al. 2006; Magalhaes et al. 1994; Starosta-Rubinstein et al. 1987), diffusion or focal hypoperfusion in superior frontal, prefrontal, parietal, occipital cortices, in temporal gyri, caudatus and putamen with single photon emission computed tomography (SPECT) studies (Piga et al. 2008), and decreased cerebellar, striatal and cortical and thalamic regional cerebral metabolic rate of glucose consumption (rCMRGlc) with positron emission tomography (PET) (Kuwert et al. 1992; Hawkins et al. 1987). In the current study, we collected both structural and resting-state imaging data in WD patients and normal controls, aiming to provide additional information other than structural changes about how WD will affect brain.

In the last two decades, reliable and easy-to-perform resting-state functional magnetic resonance imaging (R-fMRI) has attracted more and more attention in the diagnosis of brain activities since Biswal and his colleagues (Biswal et al. 1995) first reported that spontaneous low-frequency oscillations (LFO; 0.01–0.08 Hz) of blood oxygen level-dependent (BOLD) signal were physiologically informative. Amplitude of low-frequency fluctuations (ALFF), characterizing the local brain features of R-fMRI data, is one of the most frequently used approaches that quantify the regional neural synchronous activity by assessing the amplitude of power spectrum of LFO (Zang et al. 2007). The ALFF analysis has been used to detect brain abnormalities in a spectrum of diseases, such as the attention-deficient hyperactivity disorder (Zang et al. 2007), Alzheimer’s disease (He et al. 2007), and posttraumatic stress disorder (Yin et al. 2011). While resting-state PET/SPECT directly measures the metabolism of local brain areas, the ALFF measures the deviation of the BOLD signal and may provide extra information about how disease affects brain.

In the present study, we utilized ALFF of R-fMRI to examine WD-related abnormal changes in LFO amplitudes. We computed ALFF at the slow-5 (0.01–0.027 Hz) and slow-4 bands (0.027–0.073 Hz) to evaluate if the WD-related deficits were frequency-specific. Stronger ALFF at slow-5 tended to be present in the cortical structures, and stronger slow-4 tended to be present in the subcortical structures (Zou et al. 2010). Moreover, several diseases have been shown to be band specific (Hoptman et al. 2010; Wang et al. 2014). Since other frequency bands, e.g. slow-6 (0–0.01 Hz), slow-3 (0.073–0.198 Hz), and slow-2 (0.198–0.25 Hz), may mingle with very low frequency drift, the white matter (WM) signals, and high-frequency physiological noises, they were not analyzed (Biswal et al. 1995; Zou et al. 2010). We also investigated the associations between ALFF and biochemical measures, e.g. 24-h urinary copper excretion (Cu) and serum ceruloplasmin (CP), aiming to confirm the relationship between ALFF measures and clinical diagnosis.

## Method

### Subjects

Twenty-eight neurological WD patients (18 males), with 21.43 ± 3.56 years (mean ± s.d.), were recruited from the Institute of Neurology at Anhui University of Chinese Medicine, Hefei, China. The mean duration of illness was 6.67 (± 1.46, s.d.) years when they participated the study. Neurological WD patients were hospitalized and were intravenously administered sodium dimercaptopropanesulfonate (DMPS) at a dose of 15–20 mg/kg throughout the course of the treatment to promote the urinary excretion of copper. Each treatment course consisted of DMPS therapy for 6 consecutive days, followed by a withdrawal period of 2 days to promote tolerance. Every patient received repeated DMPS therapy for 8 to 10 courses every year. All patients demonstrated neurological improvements and decreased urinary copper excretion after 6 courses but experienced worsening of symptoms after 6 months to 1 year post DMPS therapy. All neurological WD patients who participated in this study didn’t receive any treatments during the past 6 months before this study. They began the treatment after the procedure of diagnosis and scanning. The diagnosis was made based on clinical symptoms (e.g., presentation of extrapyramidal symptoms and signs, with dysarthria and tremor, and remarkable impairment of liver function), the presence of the Kayser-Fleischer Ring (the KF ring), and abnormal copper metabolism (decreased levels of serum ceruloplasmin: CP < 20 mg/dL; increased 24-h urinary excretion of copper: 24-h urinary Cu > 1.6 μmol/day) (European Association For The Study Of The Liver 2012). The average 24-h urinary Cu was 2.42 ± 1.48 μmol/day (mean ± s.d.) and the average CP was 6.15 ± 2.51 mg/dL (mean ± s.d.) based on the group of neurological WD patients. Twenty-seven normal controls (NC; 15 males), aged 23.41 ± 2.65 years (mean ± s.d.), were recruited from the local community. Subjects (patients and normal controls) with history of head injury, neurological disorders, or concomitant medical disorders were excluded. All subjects, with written informed consents, were scanned within 24 h of initial contact. This study was approved by the Medical Research Ethics Committee of Anhui Medical University. The characteristics of all subjects were summarized in Table 1.

**Table 1:** Characteristics of the participants

	WD (*N* = 28)	NC (*N* = 27)
Gender (male/female)	18/10	15/12
Age (years)	16–27 (21.43 ± 3.56)	17–31(23.41 ± 2.65)
Education (years)	8–14 (10.19 ± 1.33)	9–15 (11.02 ± 2.52)
Handedness	28 right-handed	27 right-handed
WD duration (years)	4–9 (6.67 ± 1.46)	
WD types	neurologic	
The KF ring	28 WD with the KF ring	
24-h urinary Cu (μmol/day)	1–6 (2.42 ± 1.48)(*N* = 19)	
CP (mg/dL)	3.43–13.02 (6.15 ± 2.51)(*N* = 19)	

*WD* Wilson’s disease, *NC* Normal controls, *N* Number, *KF* Kayser-Fleischer, *Cu* Copper, *CPN* Ceruloplasmin

### MRI data acquisition

All subjects were scanned on a GE HDxt (General Electric Medical Systems, Milwaukee, WI, USA) 3.0 T magnetic resonance scanner at the first-affiliated hospital of Anhui Medical University. Resting-state fMRI was obtained using an 480-s EPI (echo planar imaging) pulse sequence: TR (repetition time) = 2000 ms, TE (echo time) = 30 ms, flip angle =90°, field of view =240 × 240 mm^2^, voxel size =3.75 × 3.75 × 3.75 mm^3^, matrix =64 × 64, number of slices =39, slice thickness = 3 mm, gap =0.8 mm. For structural imaging, a 3-dimensional spoiled gradient-recalled acquisition (3D–SPGR) technique was used: TR = 7.012 ms, TE = 2.876 ms, TI = 900 ms, flip angle =8°, field of view =256× 256 mm^2^, voxel size =0.94 × 0.94 × 1.2 mm^3^, matrix =256 × 256, slice thickness = 1 mm without gap, 166 slices.

### Data preprocessing

The preprocessing was carried out using Data Processing Assistant for Resting-State fMRI (DPARSF, http://www.restfmri.net/) (Chao-Gan and Yu-Feng 2010), which is based on Statistical Parametric Mapping (SPM8, http://www.fil.ion.ucl.ac.uk/spm/) and Resting-State fMRI Data Analysis Toolkit (REST, http://www.restfmri.net/) (Song et al. 2011). First ten volumes were discarded to allow MRI signal to reach a steady state and the subjects to get used to the scanner environment. The remaining fMRI images were slice-acquisition and head-motion corrected with a least-square approach and a six-parameter spatial transformation. Three WD patients and one normal control were excluded according to the criteria that head motion was restricted to less than 2 mm of displacement or 2 degrees of rotation in any directions. Six motion parameters, the cerebrospinal fluid (CSF), and the white matter signals were used as nuisance covariates to reduce the effects of head motion and non-neuronal BOLD fluctuations (Biswal et al. 1995; Zou et al. 2010). All functional data were normalized to the standard SPM8 Montreal Neurological Institute (MNI) template by applying the transformation parameters obtained from the structural images to those corrected images, and then resampled to 3-mm^3^ voxels. The resultant normalized functional images were spatially smoothed with a Gaussian kernel of 4-mm full width at half maximum (FWHM) and removed of linear trends.

### ALFF analysis

We used REST software to calculate ALFF (Zang et al. 2007; Song et al. 2011). The time series for each voxel was first band-pass filtered (0.01–0.027 Hz for slow-5 band, and 0.027–0.073 Hz for slow-4). The filtered time series was fast Fourier transform (FFT) transformed to obtain power spectrum in the frequency domain (parameters: taper percent 0, FFT length shortest). The square root of the power spectrum was computed and averaged across pre-defined frequency intervals at each voxel. This averaged square root was taken as the ALFF at a particular frequency band. To reduce the effects of participants’ variability, ALFF of each voxel for each participant was further divided by the global mean ALFF (Zang et al. 2007). The global mean ALFF value was calculated for each participant within a group gray matter (GM) mask obtained by selecting a threshold of 0.2 on the mean GM map of all 51 subjects.

### Structural image analysis

The loss of GM in WD may confound the ALFF analysis. To identify the brain regions with GM loss, we performed a voxel-based morphometry (VBM) analysis for structural images (http://www.fil.ion.ucl.ac.uk/spm). Briefly, individual structural images (3D T1-weighted anatomical images) were co-registered to the mean functional images after motion correction using a linear transformation (Collignon et al. 1995). The transformed structural images were then segmented and spatially normalized into GM, WM, and cerebrospinal fluid in the MNI space by using a unified segmentation algorithm (Ashburner and Friston 2005). Individual GM maps were further modulated to compensate for the effect of spatial normalization using both linear and nonlinear methods. The GM images underwent a spatial smoothing using 4-mm full width at half maximum (FWHM) Gaussian kernel. The resultant images were used to identify the brain regions with GM loss. Moreover, these maps were also taken into the following statistical analysis to examine the effects of GM atrophy on the functional results. In the study, we utilized the mean GM map (threshold =0.2) to generate a group-based GM mask and used this mask for analyzing ALFF differences between the normal and WD groups.

### Statistical analysis

A voxel-based two-sample t-test was applied on the smoothed GM intensity maps to determine the results of GM loss in the WD patients. We performed a two-way repeated-measure analysis of variance (ANOVA) on a voxel-by-voxel basis with group (WD patients and normal controls) as a between-subject factor and frequency band (slow-4 and slow-5) as within-subject factors in which the GM intensity maps were entered as covariates. Monte Carlo simulation was utilized to correct for multiple comparisons by using the Resting-State fMRI Data Analysis Toolkit (version 1.8) (Song et al. 2011). A corrected significance level of *P =* 0.05 was obtained by a combined threshold of *P =* 0.01 for each voxel and an extent threshold of 66 voxels (cluster size >1782 mm^3^). The correlation analyses were conducted after GM correction. Spearman’s correlation coefficient was used to study the association between LFO amplitudes and biochemical parameters, i.e., Cu ( μmol/day) and CP (mg/dL).

## Results

### GM volume

**Fig. 1:**
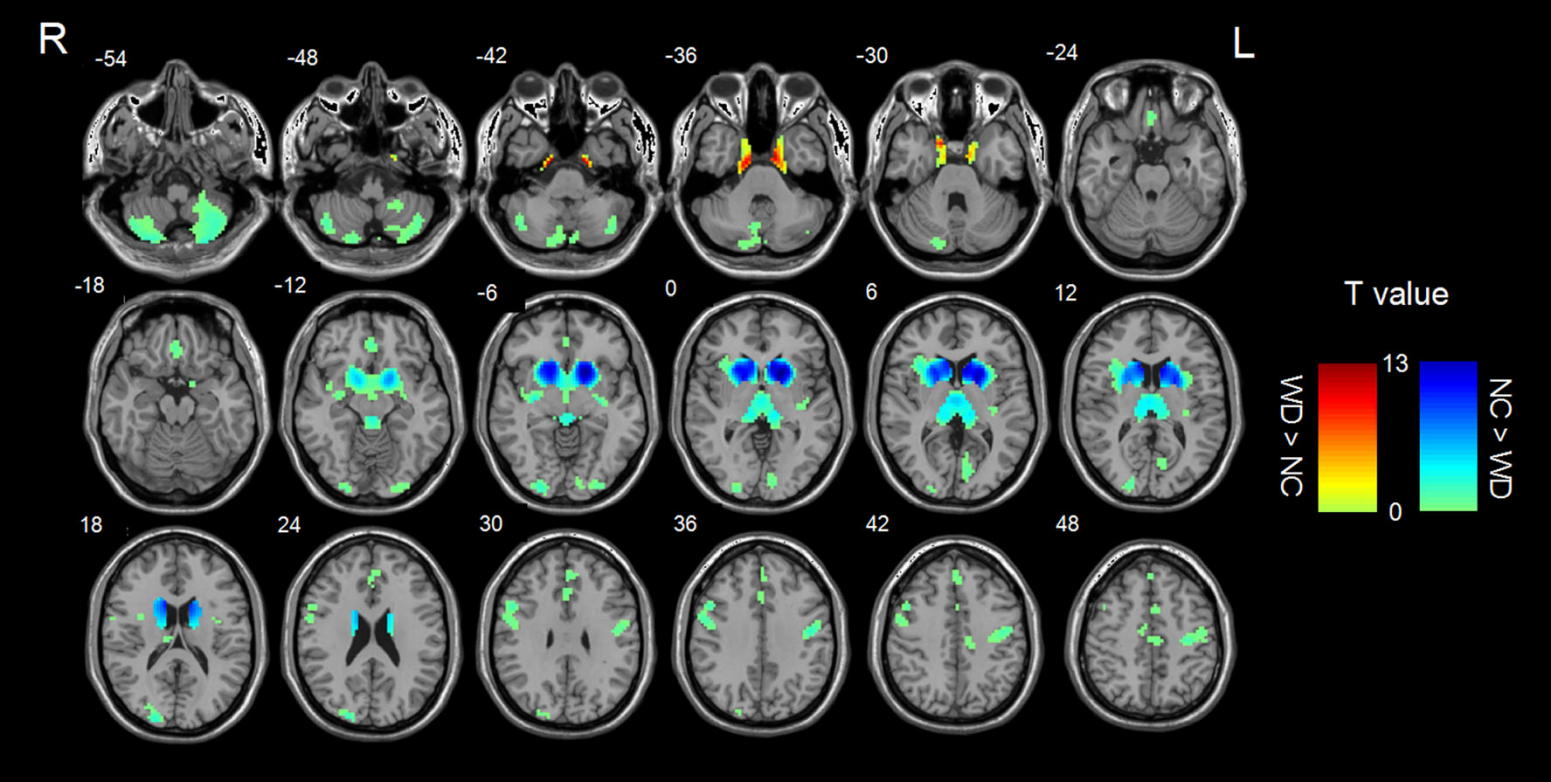
GM changes in WD patients as compared to normal controls (two-sample t-tests)

VBM analysis revealed decreased GM volume in the basal ganglia, bilateral cerebellum, right superior and left middle occipital gyrus, right middle frontal gyrus, bilateral medial frontal gyrus, left precentral gyrus, left paracentral lobule, and increased GM volume in bilateral parahippocampal gyrus (Fig. 1, Table 2).

**Table 2:** Brain regions showing significant GM differences between patients and controls

Brain regions	Voxels	MNI coordinates (mm)			T value
		x	y	z	
WD < NC					
Basal ganglia	2281	-18	12	0	-12.39
Left Cerebellum	291	-27	-81	-57	-5.06
Right Cerebellum	277	27	-81	-54	-4.07
Left precentral gyrus	187	-45	-12	36	-4.38
Left paracentral lobule	135	0	-21	54	-4.06
Right middle frontal gyrus	184	51	12	33	-4.21
Right medial frontal gyrus	98	3	36	-15	-3.78
Left medial frontal gyrus	127	-3	42	27	-3.49
Right superior occipital gyrus	176	21	-93	15	-4.53
Left middle occipital gyrus	155	-30	-93	-6	-3.46
WD > NC					
Left parahippocampal gyrus	107	-12	-6	-39	5.27
Right parahippocampal gyrus	112	15	-9	-39	5.56

Note: x, y, z, coordinates of primary peak locations in the MNI space; T, statistical value of peak voxel showing ALFF differences between two groups. *P* < 0.05, corrected for multiple comparisons

### ALFF (slow 5: 0.01–0.027 Hz, slow 4: 0.027–0.073 Hz) changes in Wilson’s disease

**Fig. 2:**
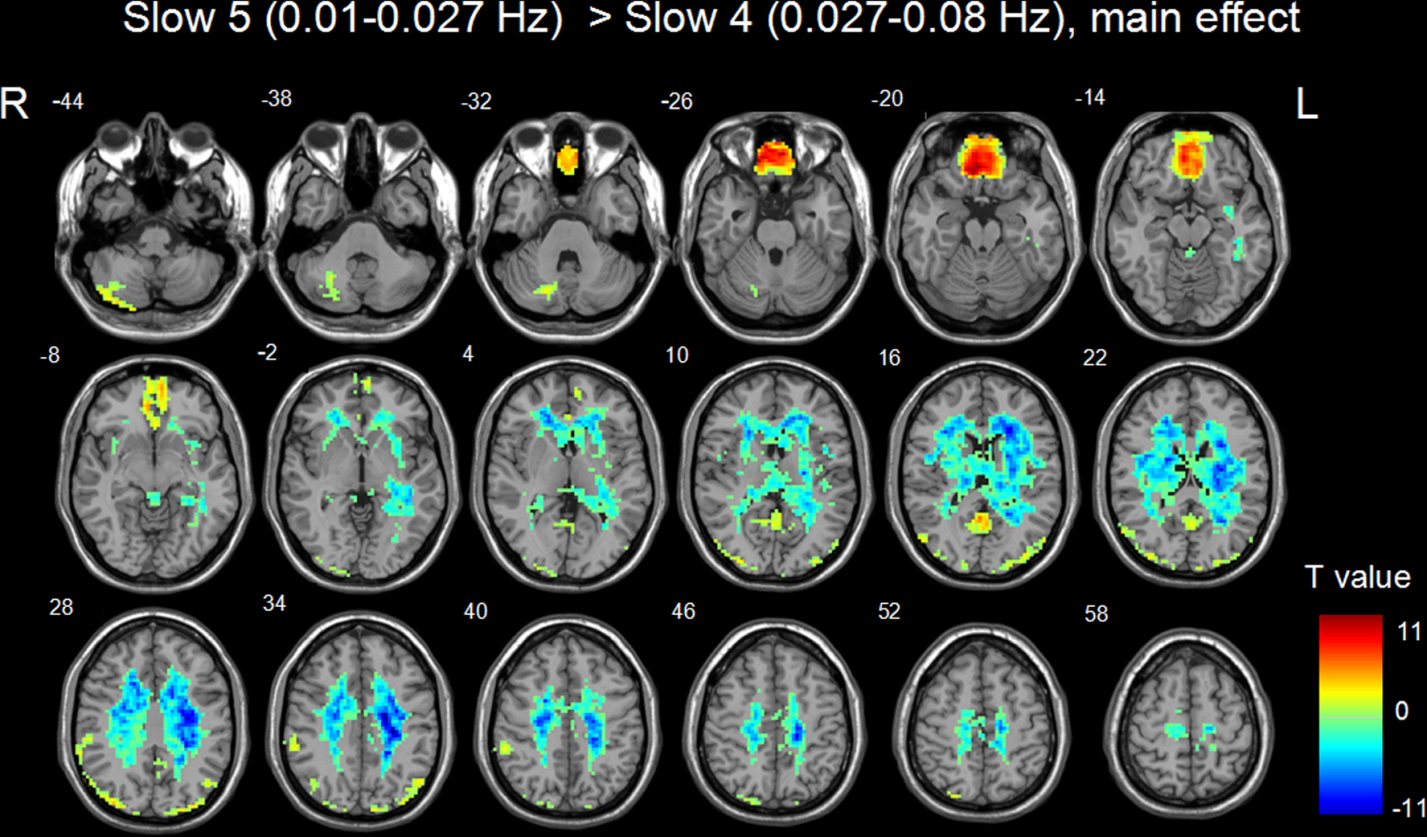
Frequency-specific ALFF deficits. *Hot color* represents greater ALFF in the slow-5 band than in the slow-4 band, whereas *blue color* represents lower ALFF

**Fig. 3:**
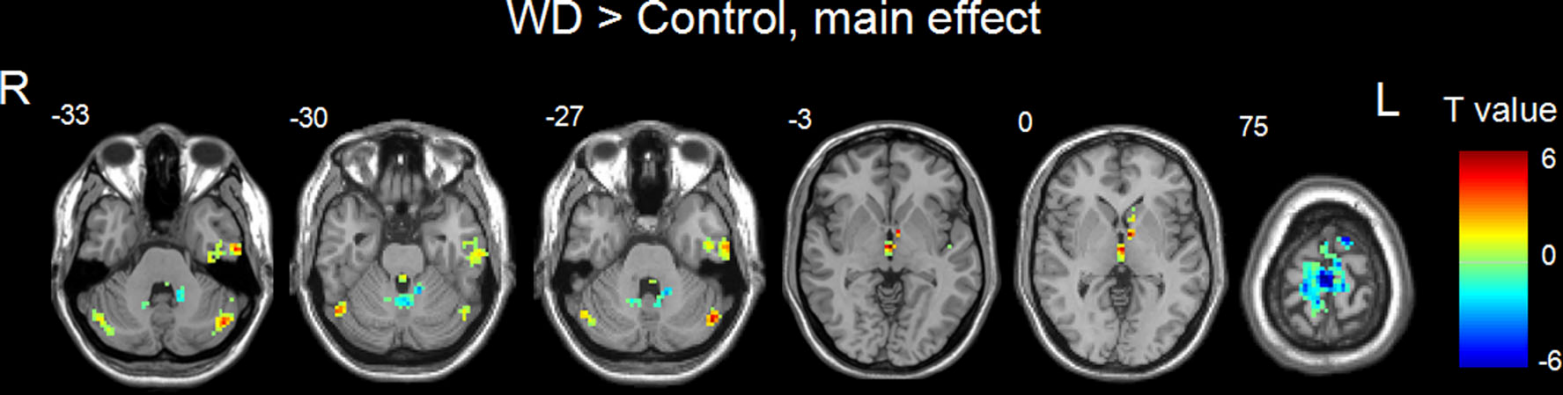
Regions with ALFF difference between WD and normal groups. *Hot color* represents higher ALFF in the WD group than in the control group, whereas *blue color* represents lower ALFF

**Fig. 4:**
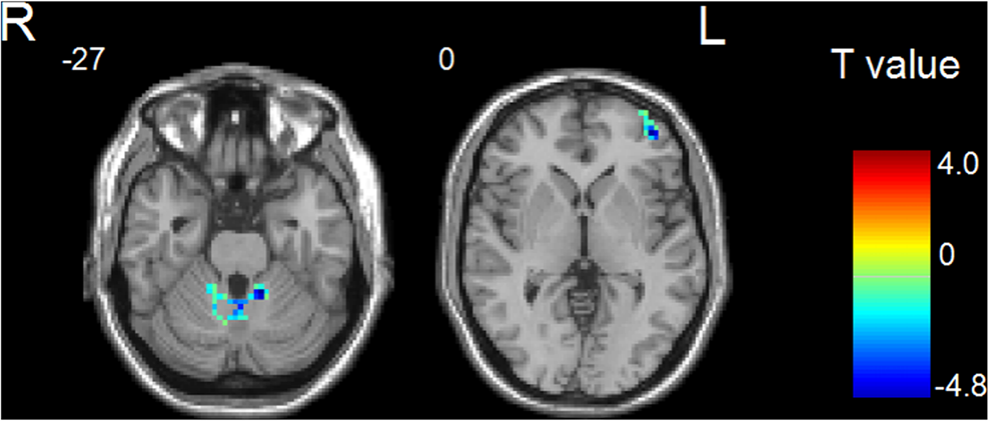
Areas showing greater difference in slow-5 than in slow-4 between WD and normal groups

Figures 2 and 3 showed the main effects from the two-way repeated-measure ANOVA. Brain regions showing a significant main effect for frequency band were identified in the medial frontal gyrus (Fig. 2, slow-5 > slow-4), inferior temporal gyrus, insula, basal ganglia, hippocampus/parahippocampal gyrus, and thalamus bilaterally (Fig. 2, slow-5 < slow-4). Figure 3 shows brain regions with a main effect of group, including the cerebellum posterior lobe, inferior temporal gyrus, brain stem, basal ganglia (patient > control), cerebellum anterior lobe and medial frontal gyrus (patient < control). We observed significant interaction between frequency band and group in the cerebellum and left middle frontal gyrus (Fig. 4). Further post-hoc *t*-test revealed that ALFF abnormalities were greater in the slow-5 than in the slow-4 band.

### Correlation analyses

**Fig. 5:**
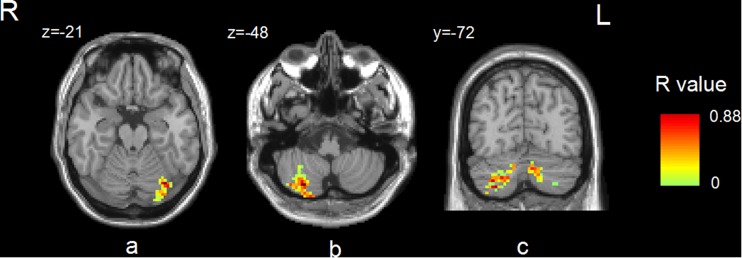
Areas showing correlation between **a** Cu ( μmol/day) and slow-5, **b** CP (mg/dL) and slow-5, **c** CP (mg/dL) and slow-4

The associations between LFO amplitudes and biochemical parameters, i.e., Cu and CP were analyzed. The Cu level was found to be positively correlated with Slow-5 in the left cerebellum (Fig. 5a), but not with Slow-4. Positive correlations were also found between CP and ALFF values in the right cerebellum in Slow-5 (Fig. 5b) and in the bilateral cerebellums in Slow-4 (Fig. 5c).

## Discussion

In the present study, we examined changes in ALFF in WD patients at different frequency bands (slow-5: 0.01–0.027 Hz; slow-4: 0.027–0.073 Hz) with GM correction. We found a general brain atrophy in WD patients, and widespread differences in ALFF of the two bands in many brain regions. Specifically, compared to normal controls, WD patients had increased ALFF in the posterior lobe of the cerebellum, inferior temporal gyrus, brain stem, basal ganglia, and decreased ALFF in the anterior lobe of the cerebellum and medial frontal gyrus, with greater group difference in the slow-5 band than in the slow-4 band in the cerebellum and left middle frontal gyrus. Correlation analysis revealed consistently positive correlations between Cu, CP and ALFFs in the cerebellum.

### Changes in GM volume

The VBM analysis showed that the decrease of GM volumes was most significant in the basal ganglia and were also found in cerebellum, middle and medial frontal gyrus, superior and middle occipital gyrus, precentral and paracentral lobule. The results were consistent with previous MRI studies investigating structural changes in WD patients (Magalhaes et al. 1994; Starosta-Rubinstein et al. 1987), which showed that most obvious lesions were seen in the basal ganglia, and generalized brain atrophy was also common. Because the GM atrophy may lead to artificial reduction in regional ALFF results, all the functional results in the current study have been adjusted with GM correction.

### Abnormal ALFF activity in WD patients

Compared to normal controls, WD patients had increased ALFF in the posterior lobe of the cerebellum, inferior temporal gyrus, brain stem, basal ganglia, and decreased ALFF in the anterior lobe of the cerebellum and medial frontal gyrus. These results suggest specific spontaneous neural activity in different brain regions in WD may have different characteristics from healthy people’s. These regions showing abnormal LFO amplitude echoed with the neurological symptoms of WD disease. Previous observations have documented neurological symptoms in WD patients in the form of parkinsonism (cogwheel rigidity, bradykinesia or slowed movements and a lack of balance are the most common parkinsonian features), masked facial expressions, ataxia (lack of coordination) or dystonia (twisting and repetitive movements of part of the body) (Lorincz 2010). Since lesions of the basal ganglia and cerebellum will lead to motor deficits in animals and humans (Albin et al. 1989; Chesselet and Delfs 1996; Jueptner and Weiller 1998; Barinaga 1996), the abnormal ALFFs observed in basal ganglia and cerebellum are possibly responsible for the motor control or movement-related impairment in WD patients. Starosta-Rubinstein et al. evaluated 31 WD patients with detailed neurologic and medical examinations and found dystonia and bradykinesia correlated with putamen lesions, and dysarthria correlated with both putamen and caudate lesions (Starosta-Rubinstein et al. 1987). On the other hand, the abnormalities in the brainstem may result in changes in sensation, muscle weakness, swallowing and speech difficulty, and co-ordination problems in WD patients (Ertekin et al. 2000; Carmichael et al. 1965). Moreover, the cognition problems (i.e. impulsivity, impaired judgement, promiscuity, apathy and executive dysfunction, slow thinking, and memory loss etc.) and psychiatric problems (i.e. depression, anxiety and psychosis etc.) due to WD (Ala et al. 2007; Lorincz 2010) might result from the joint abnormal functions of the medial frontal gyrus, inferior temporal gyrus and other subcortical regions such as basal ganglia (Ridderinkhof et al. 2004; Chan et al. 2001; Kempton et al. 2011).

In addition, our results showed greater ALFF values for slow-5 band than slow-4 band in the medial frontal gyrus, and greater ALFF values for slow-4 band than slow-5 band in the inferior temporal gyrus, insula, basal ganglia, hippocampus/parahippocampal gyrus, and thalamus, supporting the results by Zuo et al. (2010) revealing that stronger ALFF at slow-5 tended to be present in the cortical structures, and slow-4 tended to be in the subcortical structures. Specifically, we observed that the ALFF abnormalities in the cerebellum and middle frontal gyrus were greater in the slow-5 than in the slow-4 band, indicating band-specific abnormal ALFF in WD patients.

### Correlations between LFO amplitudes and biochemical parameters

To study the associations between LFO amplitudes and biochemical measures could help us better understand the relationship between ALFF values and diagnosis of WD. The results show positive correlations between Cu and slow-5, CP and ALFFs at both frequency bands (slow-4 and slow-5) in the cerebellum. It has been shown that most copper is deposited in the basal ganglia of WD, which normally participate in the coordination of movement. Damage to these areas, by Fenton chemistry, produces the neuropsychiatric symptoms seen in Wilson’s disease (de Bie et al. 2007). However, we didn’t find the correlations between Cu, CP and ALFF in the basal ganglia. This may be because all of our ALFF results have been adjusted with GM correction and WD patients have a large GM volume loss in the basal ganglia region as indicated by the VBM analysis. As a result, the information revealed by ALFF is limited in the basal ganglia. By contrast, WD patients showed strong abnormal ALFF activity in large regions of the cerebellum, which area also plays an important role in motor control. Thus, the correlations found between ALFF and biochemical measures such as Cu and CP in the cerebellum indicate the WD related changes in the movement function.

Future studies could consider taking patients with hepatic WD as the control, which would be optimal to compare the brain activity changes in the neurological WD. Moreover, it would be interesting to investigate ALFF changes before and after the treatment, and to correlate the ALFF changes with the clinical outcome. All of our WD patients were treated with DMPS, which is widely used in China for more than 30 years (Chen et al. 2016; Yang and Cheng 2002; Wang et al. 2003). We noted that clinical practice with WD patient in Europe and the United States used Penicillamine instead of DMPS (European Association For The Study Of The Liver 2012). Although the general mechanisms underlying DMPS and Penicillamine might be similar (Gerhardsson and Aaseth 2016), whether our findings with ALFF abnormalities were universal characteristics of WD patient required further investigation.

## Conclusion

This study shows that WD patients had abnormal LFO amplitude in many brain regions, including the cerebellum, inferior temporal gyrus, brain stem, basal ganglia, and medial frontal gyrus, with different spatial patterns in different frequency bands, and there was a correlation between LFO amplitudes and biochemical measures in the cerebellum. Thus, the accumulation of copper profoundly impaired intrinsic brain activity and the impairments seem to be frequency-dependent. These results provide further insights into the understanding of the pathophysiology of WD.
